# Evaluation of Location-Specific Predictions by a Detailed Simulation Model of *Aedes aegypti* Populations

**DOI:** 10.1371/journal.pone.0022701

**Published:** 2011-07-25

**Authors:** Mathieu Legros, Krisztian Magori, Amy C. Morrison, Chonggang Xu, Thomas W. Scott, Alun L. Lloyd, Fred Gould

**Affiliations:** 1 Department of Entomology, North Carolina State University, Raleigh, North Carolina, United States of America; 2 Department of Entomology, University of California Davis, Davis, California, United States of America; 3 Odum School of Ecology, University of Georgia, Athens, Georgia, United States of America; 4 Fogarty International Center, National Institutes of Health, Bethesda, Maryland, United States of America; 5 Department of Mathematics and Biomathematics Graduate Program, North Carolina State University, Raleigh, North Carolina, United States of America; Duke-National University of Singapore, Singapore

## Abstract

**Background:**

Skeeter Buster is a stochastic, spatially explicit simulation model of *Aedes aegypti* populations, designed to predict the outcome of vector population control methods. In this study, we apply the model to two specific locations, the cities of Iquitos, Peru, and Buenos Aires, Argentina. These two sites differ in the amount of field data that is available for location-specific customization. By comparing output from Skeeter Buster to field observations in these two cases we evaluate population dynamics predictions by Skeeter Buster with varying degrees of customization.

**Methodology/Principal Findings:**

Skeeter Buster was customized to the Iquitos location by simulating the layout of houses and the associated distribution of water-holding containers, based on extensive surveys of *Ae. aegypti* populations and larval habitats that have been conducted in Iquitos for over 10 years. The model is calibrated by adjusting the food input into various types of containers to match their observed pupal productivity in the field. We contrast the output of this customized model to the data collected from the natural population, comparing pupal numbers and spatial distribution of pupae in the population. Our results show that Skeeter Buster replicates specific population dynamics and spatial structure of *Ae. aegypti* in Iquitos. We then show how Skeeter Buster can be customized for Buenos Aires, where we only had *Ae. aegypti* abundance data that was averaged across all locations. In the Argentina case Skeeter Buster provides a satisfactory simulation of temporal population dynamics across seasons.

**Conclusions:**

This model can provide a faithful description of *Ae. aegypti* populations, through a process of location-specific customization that is contingent on the amount of data available from field collections. We discuss limitations presented by some specific components of the model such as the description of food dynamics and challenges that these limitations bring to model evaluation.

## Introduction

The mosquito *Aedes aegypti* is the major vector of dengue virus. This virus causes approximately 50 million cases of dengue fever each year [1], and sporadic epidemic outbreaks can overwhelm health systems in affected countries [2]. The epidemiology of dengue is complicated due to a number of factors including the existence of 4 dengue serotypes [3] and variation in the population dynamics of *Ae. aegypti* [4]. Because there is no vaccine for dengue or drugs to alleviate symptoms, efforts to suppress dengue have relied on vector control [5]. The impact of vector control programs based on conventional technologies is often difficult to predict [5], [6]. Assessing the potential of new control methods based on manipulation of *Ae. aegypti* genetics [7], [8] is no less challenging. Any such predictions must account for interactions between the biology and behavior of the vector, the pathogen and the human host [9].

Mathematical models that include *Ae. aegypti* population dynamics are essential for this task. One model, CIMSiM [10], [11], includes details of the *Ae. aegypti* biology and dynamics, but lacks spatial dimensions, population genetics, and stochastic processes. Another model which is spatially explicit, includes fewer biological details than CIMSiM and lacks genetics [12]. To address the need for an *Ae. aegypti* model that includes both ecological and genetic realism we developed the Skeeter Buster model [13], a spatially explicit, weather-driven, stochastic simulation of *Ae. aegypti* population dynamics and genetics [14].

Skeeter Buster is based on many components of the previously developed CIMSiM model [10], [11], and as such, includes a detailed representation of *Ae. aegypti* biology. Further levels of model complexity were added to Skeeter Buster, including stochasticity and a fine-scale spatial structure (down to the level of individual containers). As a result, Skeeter Buster is a complex ecological model of *Ae. aegypti* populations, including over 100 parameters as well as detailed inputs of weather data, container distribution and nutritional resource availability. In a previous study, Magori *et al.* [15] described the details of this model's parameters and procedures, including default values of all parameters based on field and lab studies found in the literature. A quantitative assessment of uncertainties in model predictions arising from uncertainties in parameter value estimates and from model stochasticity was recently published [16].

For this model to be useful as a tool to guide and assess the operational development of control strategies, we must first be confident that it can accurately describe specific *Ae. aegypti* populations in targeted locations. Skeeter Buster is designed to be customized for targeted locations, using specific climatic data as well as mosquito habitat information. The ability of the model to simulate the dynamics of the population in the location of interest is expected to depend on local information about distribution of larval development sites and pupal production from specific categories of sites (e.g. buckets, tires), but how much of this local information is needed for accurate model predictions had not been determined.

The purpose of this study was, therefore, to test the ability of Skeeter Buster to reflect the field population dynamics of *Ae. aegypti* in two separate locations: the tropical city of Iquitos, Peru and the temperate city of Buenos Aires, Argentina.

We carried out the Iquitos analysis based on historical weather data for the city as well as data obtained from prior field surveys in this city on (1) the distribution and characteristics of water-filled containers in the city (used as model input), and (2) a detailed, stage-specific, quantitative account of the local mosquito population, at the level of individual houses and individual containers. We describe here the details of these two data sets, and how they are used to customize the model specifically for the Iquitos location, including notably a calibration of container productivity for various types of containers. We then present the results of the customized Skeeter Buster and test the predictions against independent data from field studies, showing that the model accurately describes several aspects of *Ae. aegypti* population dynamics in Iquitos.

We then examine the applicability of the model to Buenos Aires to assess the level of detail in *Ae. aegypti* population dynamics that Skeeter Buster can simulate where the available field data are more limited. We conclude this study by discussing the importance of data availability as well as identifying the corresponding model complexities that present challenges for future model applications.

## Materials and Methods

### Ethics statement

The Iquitos survey protocol was approved by the University of California, Davis (Protocol 2220210788-4(994054), Instituto Nacional de Salud, and Naval Medical Research Center (Protocol #NMRCD.2001.0008 [DoD 31574])) Institutional Review Boards in compliance with all Federal regulations governing the protection of human subjects. All subjects provided written informed consent.

### Iquitos, Peru: Study area and survey methods

Iquitos, Peru (3 44′ S, 73°15′ W, 120m above sea level) is a city of approximately 380,000 people located in northeastern Peru in the Amazonian rainforest. Iquitos constitutes a prime study site for *Ae. aegypti* populations because of its relative isolation, with no land-based connection with other population centers [17]. The climate is equatorial, characterized by year-round high humidity and high temperatures. The average maximum daily temperature (with 5%–95% range) is 32.2°C (29°C –35°C), the average minimum daily temperature is 21.4°C (19°C –23°C), and the average annual rainfall is 2,878 mm. Precipitation occurs frequently all year with no marked rainy or dry seasons. Other studies provide further details on the environmental and demographic characteristics of this city [18]–[22].

Since January 1999, extensive surveys of mosquitoes have been carried out across the city. The detailed protocols for these surveys have been described by Morrison et al. [22]. In short, these surveys consist of visits to individual households in various Iquitos neighborhoods. In each house, adult mosquitoes were collected using backpack aspirators and containers were examined for the presence and abundance of *Ae. aegypti* pupae (individually counted) or larvae (number visually estimated, 0, 1–10, 11–100, >100). Each container was measured and described according to a number of characteristics including sun exposure, location (inside or outside), presence of a lid, and filling method (manually-filled, passive-rain-filled or assisted-rain-filled). Each container was also assigned to one of 14 categories (listed here in decreasing order of pupal productivity): *plastic*, *medium storage*, *large tanks*, *tires*, *non-traditional*, *cooking*, *miscellaneous*, *flower pots*, *cans*, *bath*, *bottles*, *natural*, *wells* and *pet*. (See Tables 2 and 3 in [22] for more details.)

**Figure 1:**
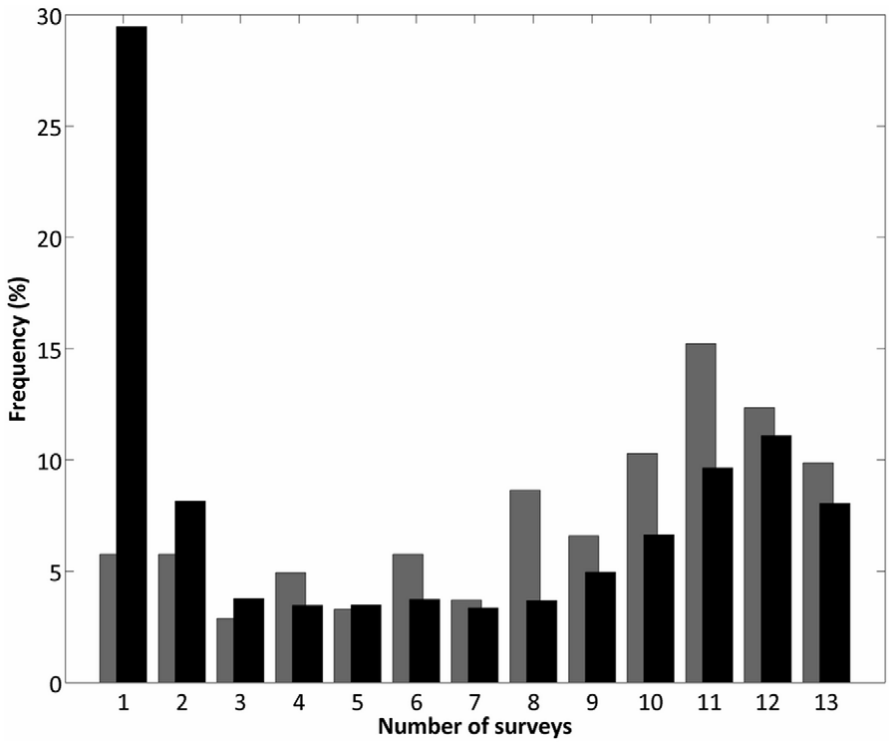
Distribution of the number of visits per house in the city of Iquitos and in our selected subset of houses. We use data from 13 distinct survey circuits during the period 1999-2003. We only consider houses that have been visited at least once. Black bars: distribution of the number of surveys for the whole city of Iquitos. Gray bars: distribution of the number of surveys in our selected 153-house subset.

Surveys were carried out along circuits that sample households across all Iquitos districts. Each circuit was completed in about 4 months, and includes single visits to approximately 6,000 houses. Prior to surveys, the entire city was geo-referenced using geographic information system (GIS) [20], [22], so that all mosquito data can be spatially located to an individual household in the city. For more detail on the geo-referencing and survey protocols, we refer the reader to the previously mentioned studies [20], [22]. In this study, we use the data collected in 13 consecutive surveys, from January 1999 to August 2003, spanning a total of 12,387 households (each circuit consisting of a different subset of those households). The average number of visits per house was 6.26, but most houses were visited either only once (29.5% of houses) or 11 times or more (29.3%) (Figure 1).

### Model customization: Location-specific model inputs

In order to apply Skeeter Buster to a specific location, we first used available data to customize model inputs to the specific environmental and ecological setting of the city of interest. In Skeeter Buster weather characteristics impact biological processes such as egg hatching, larval development rate, and daily survival probabilities of all stages [13]. We, therefore, accessed daily temperature (minimal and maximal), rainfall and relative humidity data for the city of Iquitos between 1999 and 2003. Data were obtained from the Climate Data Online (CDO) database of the National Climatic Data Center (NCDC) [23] and translated into input files for Skeeter Buster.

**Figure 2:**
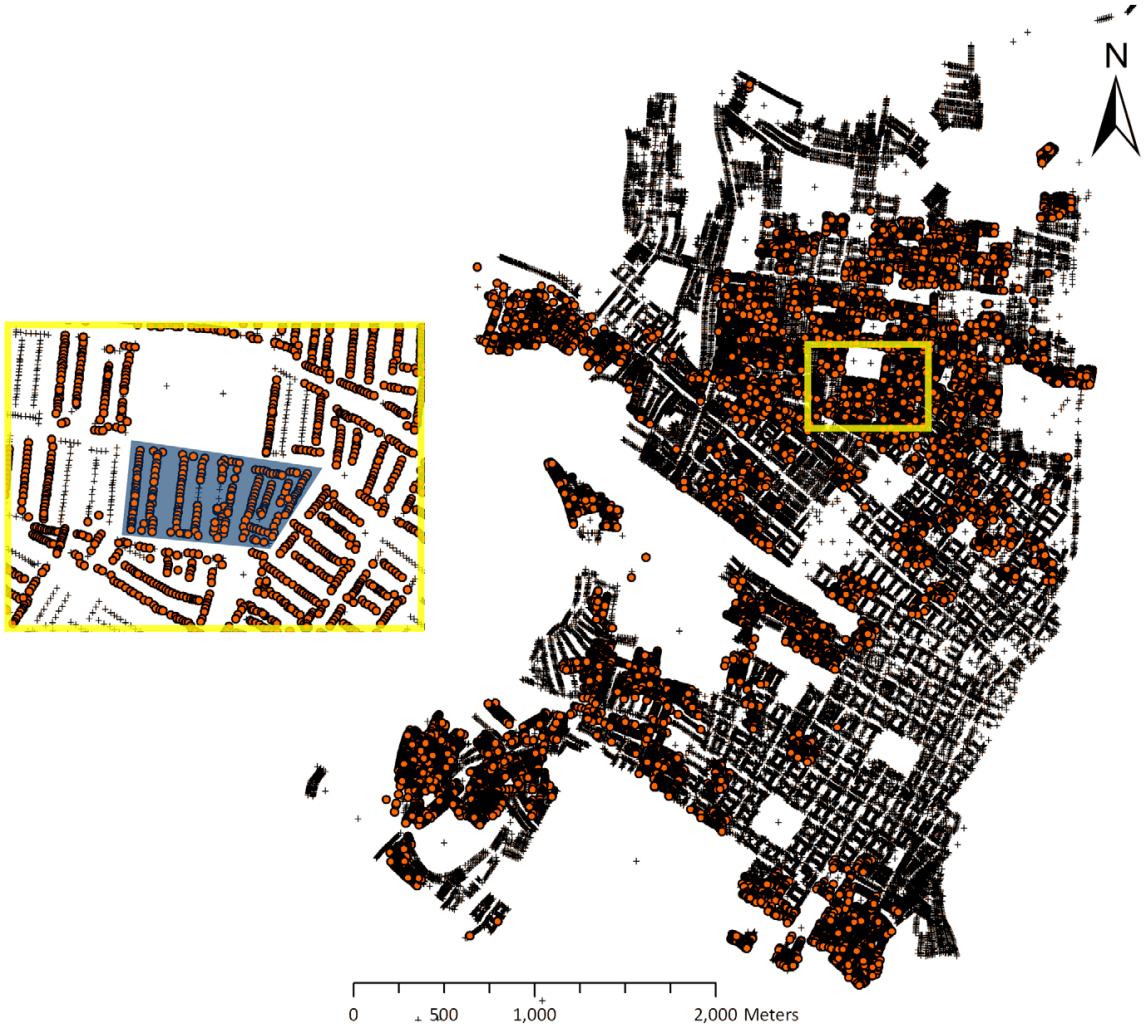
Selection of the 153-house simulation set. Right: entire city of Iquitos. Each ‘+’ symbol represents an individual house referenced in the GIS map [20], [22]. Orange circles are houses that have been included in at least one survey circuit during the period considered in this study. Inset: a zoomed-in view of part of the Maynas district (delimited in yellow). Our selected block of houses constituting the simulation set is shown as the shaded region in the inset.

Each house in a Skeeter Buster simulation is assigned a number of containers, representing potential larval development sites. Within each container the dynamics of immature cohorts are computed daily. Containers constitute potential oviposition sites for gravid females present in that house on any given day. In order to define the containers to be input into our Skeeter Buster simulations, we also used the data collected in Iquitos on the distribution and abiotic characteristics of water-filled containers. While these data included over 12,000 houses and over 290,000 individual containers, computing constraints forced us to run the model on a subset of these. We selected a set of 153 houses, arranged on a grid of 17x9 houses, as our *simulation set* (Figure 2). Two criteria were used in the selection of this particular subset of houses. First, we chose to focus on the Maynas zone of Iquitos. Maynas is a central, densely-populated area, and presents the highest levels of *Ae. aegypti* infestation [22] and highest prevalence of dengue infection [24] in the city. Second, within this district, we selected blocks of houses that had been most frequently surveyed during the 1999–2003 period. This led us to the 153-house simulation set, in which a majority of houses (63%) had been surveyed more than 8 times (Figure 1). In this subset, a total of 871 water-filled containers were found on the first survey circuit, and were used to initialize the model.

Although Skeeter Buster simulations are limited by computing power and running time, it is desirable to simulate as large an area as can be computationally managed, in order to limit the effects of stochasticity within individual houses and alleviate potential boundary effects. Because the basic 153-house simulation set was specifically selected for the repeated surveys in those houses, we chose not to extend our selection, which would have decreased the average number of surveys per house in this selected area. Instead, we copied our basic subset multiple times to define a larger grid of houses (see Supplementary Text S1, Figure S1, Figure S2 and Figure S3 for alternative options). In this study, we extend our simulated area to 4 copies of the basic subset, for a total of 612 houses and 3,484 individually-modeled containers. In each of the four instances of the 153-house block, we randomize the spatial distribution of houses on the 17×9 sub-grid, so that each of the 153 houses is present exactly 4 times in our simulation grid, but with different neighbors each time. The distribution of containers within houses is left intact, so that the same collection of containers is found in each of the four instances of a given house.

### Model calibration: Nutritional resources and calibration for Iquitos

One of the most important factors driving the development of immature *Ae. aegypti* cohorts in individual containers is the amount of nutritional resources present in each container. This amount of food is tracked for each container, and is affected by (i) a natural daily input of food, (ii) a natural decay of the available food, (iii) consumption by larval cohorts present in the container, and (iv) conversion of larvae and pupae cadavers into suitable nutritional resources [13]. The model uses the equations developed by Gilpin and McClelland [25] (see p. 366 of this reference) to track the weight gain of larval cohorts from ingested food, as well as the corresponding decline in the amount of food remaining in the container. If the amount of food in a given container is insufficient, larvae will starve for a period of time based on their available reserves, during which time they experience an increased rate of mortality. The amount of food also drives the rate at which a larval cohort gains weight, which in turn affects the time to pupation and the weight at pupation of the cohort. As a consequence, food availability affects larval density in a container through these effects on survival and development time. Of course, larval density in turn affects the amount of food available in a container. Food availability therefore constitutes in Skeeter Buster, as it does in CIMSiM, the mechanistic basis of density-dependence in the larval stages, which is generally considered an important component of the population dynamics of container-inhabiting mosquitoes [26]–[30].

Ideally, we would parameterize the containers in Skeeter Buster *a priori,* based on field information on the nutritional value of the contents of different containers. Unfortunately, very little is known about the exact origin or the precise amount of nutritional resources available in containers from natural populations. It is generally considered that microorganisms, potentially proliferating from decaying organic debris in containers, form the basis of immature mosquito nutrition [31]–[33]. There is, however, no empirical method to assess the suitability of a specific container for *Ae. aegypti* larval growth by examining only the container and the water it contains. Practically, the quality of a given container as a mosquito habitat can only be assessed based on the dynamics of immature mosquito development, measuring pupal productivity, larval development time or resistance to starvation [34]. For this reason, we follow the approach of Focks *et al.* [10] in adjusting, *a posteriori,* the average daily input of food into each container based on the pupal productivity recorded for that category of container (defined according to several container properties, see below) during the mosquito surveys. For calibration purposes, we use information collected in our *calibration set* defined as the entire set of surveyed houses excluding those selected for our simulation set. From this calibration set, data on container productivity was obtained from the 13 surveys carried out during the time period considered in this study (see Study Area and Survey Methods).

We model the daily amount *F* of food input in a given container according to the following equation:
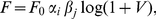
(1)where *F*_0_ is a baseline amount of food (in liver powder equivalent per unit volume), *α_i_* is a container-type-specific coefficient (*i* = 1 to 14, based on the 14 container types), *β_j_* is a container-location-specific coefficient (*j* = 1 to 2, inside or outside), and *V* is the volume of the container. Because *F*_0_, *α_i_* and *β_j_* are combined into a single multiplicative coefficient, only the relative values of *α_i_* (for different values of *i*) and *β_j_* (for different values of *j*) are important. We arbitrarily set the value of *α_i_* to 1 for large tanks and the value of *β_j_* to 1 for outside locations.

**Figure 3:**
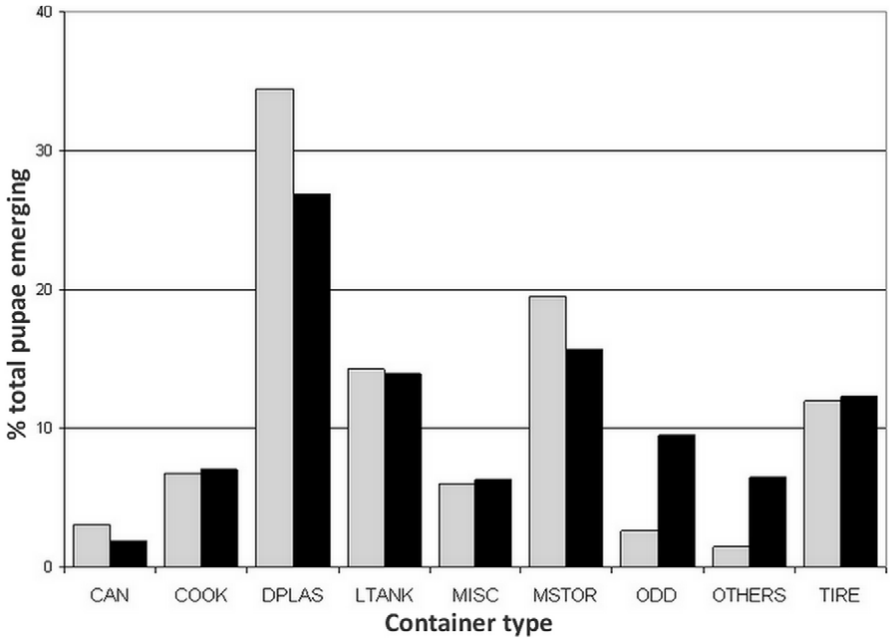
Results of the calibration of pupal productivity of different container types. The *x*-axis represents the container types defined in the Iquitos surveys [22]. Out of 14 total container types, the 8 most productive types are presented here, while the 6 remaining types are condensed into ‘Others’. The percentage of total pupae emerging from a given container type is presented for both model predictions (light gray bars) in the simulated area and field data (black bars) collected in the simulation set of houses.

In the Iquitos case, we can calculate the average pupal productivity of each container type in our calibration set. Based on these values, the coefficients *F*_0_, *α_i_* and *β_j_* are simultaneously adjusted so that the average pupal productivity per container type observed from the model matches the distribution observed in the field. The comparison between the observed productivity of each container type in our simulated area and the pupal productivity reported from the field for this same container type in the houses selected in the simulation set is presented in Figure 3.

### Spatial statistics: Comparison between simulations and empirical data

An important characteristic of a mosquito population is the level of spatial heterogeneity observed among houses, because it can potentially affect arbovirus transmission [35]. We use three statistical measures of heterogeneity and cluster size to characterize the spatial structure of simulated populations.

First, we compute the values of Moran's *I* index (Moran, 1950). This index is defined as follows:
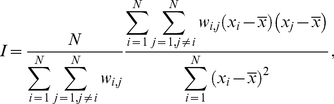
(2)where *N* is the total number of houses, *x_i_* is the number of pupae in house *i*, 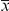
is the average number of pupae per house, and *w_ij_* is the weight between locations *i* and *j* (defined here as the reciprocal of the distance between houses *i* and *j*). Moran's *I* values range from -1 to 1, with an expected value of *–*1/(*N*-1) (*i.e.,* close to 0 for large values of *N* such as the value of *N* = 612 in this study) under the assumption of random spatial distribution. Negative values are indicative of a uniform distribution whereas positive values indicate a clustered distribution.

Next, adhering to the analysis of spatial patterns observed in the *Ae. aegypti* population in Iquitos [20], we calculate global *L_w_* and local *G_i_* statistics to characterize the existence and size of clusters of high (or low) numbers of pupae per house in our simulated populations.

*L_w_* statistics are based on *K* functions from point pattern analysis models [36]–[38] and measure the number and distribution of pairs of observations (here, the number of pupae) within a distance *d* of each other. For a given distance *d*, *L_w_*(*d*) is given by:
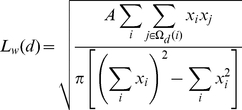
(3)where *A* is the area of the study region, *x_i_* is the number of pupae in house *i* and Ω*_d_*(*i*) is the set of houses that are within distance *d* of house *i* (excluding house *i* itself). Because, in our model setup, the presence of pupae is dependent on the presence of a house, and because houses are distributed on a regular rectangular grid (and therefore non-randomly spatially distributed), we must also compute the value of *L*(*d*) for the distribution of the houses themselves (with *x_i_* then being a dummy variable whose value equals one for each house). If pupae are randomly distributed among houses, *L_w_*(*d*) will be equal to *L*(*d*). Following [38] we calculate the increments in *L*(*d*) and *L_w_*(*d*) when *d* increases, that is, (*L_w_*(*d*) *- L_w_*(*d-1*)) *–* (*L*(*d*) *- L*(*d-1*)) for all values of *d*. An observed change in *L_w_*(*d*) greater than the change in *L*(*d*) (that is, a positive value of the above calculation) indicates that pupae are more clustered than expected given the existing pattern of houses within distance *d*.

Getis' *G_i_* statistics [20], [39] were used to measure the local distribution of pupae around house *i* to identify this particular house as a member (or not) of a cluster of pupal productivity. For a given distance *d* around house *i*, this statistic is given by:
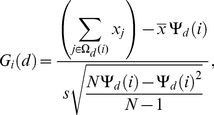
(4)where *N* is the total number of houses, 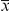 and *s* are the average and standard deviation of the number of pupae per house, and 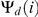 is the number of neighboring houses within distance *d* of house *i* (*i.e.* the size of the set Ω*_d_*(*i*)). If pupae are randomly distributed around house *i* the expected value of *G_i_*(*d*) is 0. Positive values (significant Z-scores above 2.575 at 0.01 confidence level [20]) indicate a cluster of high number of pupae around house *i*, negative values indicate a cluster of low number of pupae.

### Model calibration for Buenos Aires

We also consider the case of the *Ae. aegypti* population in the Mataderos neighborhood of Buenos Aires, Argentina (34.61 S, 58.37 W), a city with a temperate climate. Climatic data for the years 2001 to 2003 were obtained from the Climate Data Online (CDO) database of the National Climatic Data Center (NCDC) [23] and translated into input files for Skeeter Buster. The local *Ae. aegypti* population has been described elsewhere [40]–[42], and modeled by Otero *et al.* using another stochastic, weather-driven, spatial model that shares some assumptions with Skeeter Buster, but does not consider heterogeneity in larval development site characteristics, suitability for *Ae. aegypti* or distribution among houses [12]. Unlike Iquitos, we have no data on the types, distribution or productivities of containers, therefore Skeeter Buster cannot be customized to the same extent than in the Iquitos case, lacking a realistic description of the distribution of larval development sites in Buenos Aires. However, for model evaluation purposes, we choose to copy the grid composition and customization that was done in Iquitos. Because the two locations are ecologically very different (an isolated, medium-size city in an equatorial climate versus a neighborhood in a large metropolitan area in a temperate climate), it is likely that the Iquitos container distribution is a very poor description of the actual distribution in Buenos Aires. For the purpose of this study, this allows us to investigate the dependence of Skeeter Buster on detailed field data regarding breeding sites at the household level, and to examine the level of population dynamics prediction that can be made without such information.

We therefore set up the simulation area for Buenos Aires using the same 3,484 containers used in Iquitos, with identical characteristics and distributed identically among the 612 houses. We also use the same α*_i_* and β*_i_* coefficients to govern the amount of food present in the containers. Calibration of food amounts consists only in adjusting the *F*_0_ coefficient to match the observed overall population levels, based on surveys carried out in the Mataderos neighborhood [12], [40], [41]. These constitute the results of weekly monitoring of eggs using ovitraps deployed across the study area. We adjust population levels in the model by comparing the observed fraction of positive ovitraps each week with a daily measure of the proportion of containers in Skeeter Buster that have been oviposited into in the past 7 days, and adjusting *F*_0_ accordingly.

## Results

### Stage-specific time series in Iquitos

**Figure 4:**
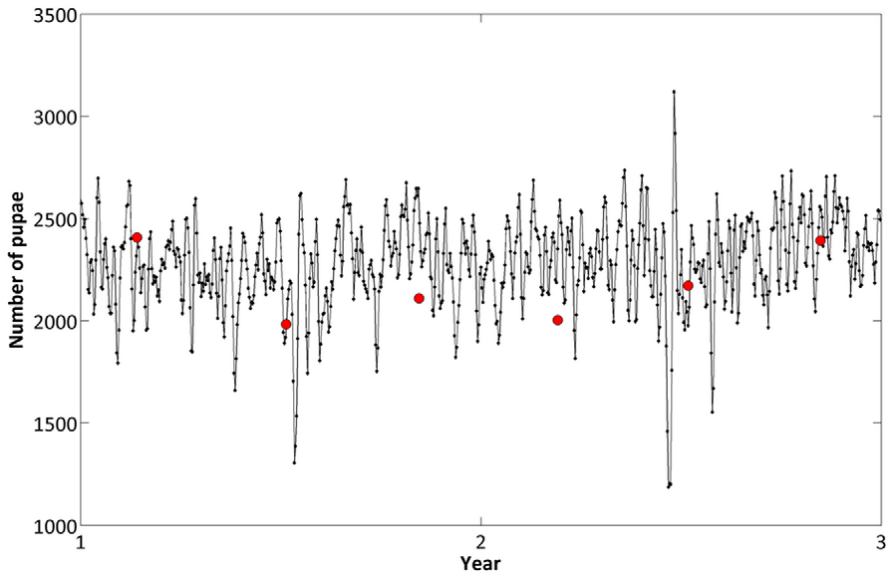
Comparison of predicted pupal time series and observed pupal counts. We compare time series of total numbers of pupae from a Skeeter Buster simulated population with values from the Iquitos survey data collected in houses forming the simulation set. Solid line presents one model outcome (with 1-year burn-in not presented). Red circles mark estimated numbers calculated from data collected during 6 separate field circuits (in 2000 and 2001) in the set of houses that corresponds to the simulated area, and adjusted to reflect our 612-house set. Note that each survey spans in reality a period of several weeks. Data points are positioned on this graph at the midpoint of each circuit.

We ran Skeeter Buster calibrated for conditions in Iquitos as described in the Methods section. All parameters of the model were set to their default values, obtained from review of previous field and lab studies of *Ae. aegypti*, and described in detail in our previous article [13]. Following the calibration process described in our Methods section, the time series of numbers of pupae predicted by the model were compared to pupal counts from surveys of the houses in the simulation set (Figure 4). Note that because each field survey spans a period of several weeks, the plotted field estimates represent averages over time (length of survey) that are not directly equivalent to daily tallies in the model output.

In the context of entomological field surveys, pupae are the only mosquito life stage that can be extensively and accurately counted, and pupal counts are considered the most reliable measure of population density for wide-scale surveys [43], [44]. The method used here ensures that the predicted overall number of pupae is in accordance with the values observed from the field. Other life stages were more difficult to extensively and accurately count during surveys; larval numbers were estimated to a range of values, not exact numbers [22] and adults were collected using backpack aspirators, but the sampling efficiency of this method is not well calibrated [45], [46]. Therefore, model predictions of the dynamics of life stages other than pupae cannot be similarly, reliably evaluated.

### Spatial structure of the Iquitos population

An important characteristic of the local population is the level of spatial heterogeneity observed among households. The Moran's *I* index can be used to test the existence of non-random spatial distribution (clustering) at the population scale. Calculations of Moran's *I* for the number of pupae per house on 20 replicated simulations of our 612-house grid showed index values ranging from −0.0011 to 0.0060 (average  = 0.0017) with no individual value significant at the 0.05 level (Z-scores ranging from −1.38 to 1.51). This indicates that no significant deviation from random distribution of pupae among houses can be detected at the scale of our simulated area. Calculations of the same Moran's *I* from data collected in 13 circuits in our selected block reveal values ranging from −0.01 to 0.01, with no individual value significant at the 0.05 level (Z-scores ranging from −0.4 to 1.7), confirming a similarly random distribution of pupae among house in this subset of the Iquitos population.

A detailed analysis of the spatial distribution of *Ae. aegypti* pupae and adults in the city of Iquitos has been previously published [20] and is based on data collected in the Maynas neighborhood where our simulation set of houses is located. This analysis examined variation among houses in number of pupae produced, and concluded that, while houses can differ in their productivity, there was an absence of clustering of high-producing houses beyond 30 meters from an individual household for adult mosquitoes, and beyond 10 meters for immature stages. While the absence of clustering detected from the model by calculations of Moran's *I* is consistent with the observed absence of large clusters in the Iquitos population, other statistics are needed to investigate the size of potential local clusters in the simulated population. Note that distances in the model can only be measured as a number of houses, or number of cells between two locations on the grid. These distances can be translated into actual geographic distances by multiplying by the average distance between houses in Iquitos, which is on the order of 5 to 10 meters.

**Figure 5:**
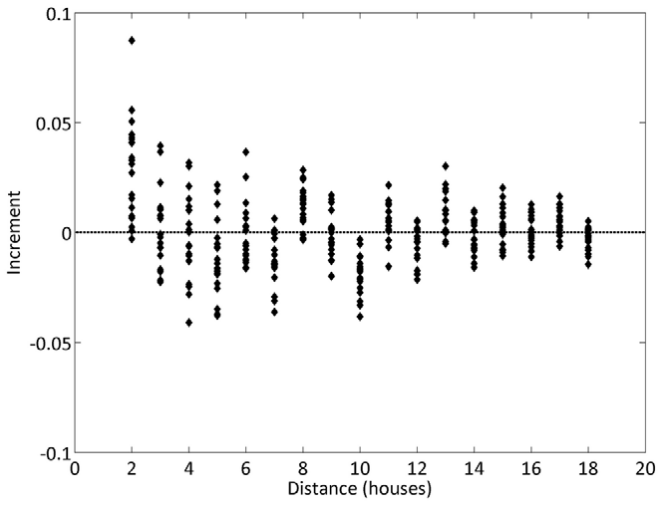
Measures of clustering in a Skeeter Buster simulated population using point-pattern analysis *L* statistic. We calculate *L* values for 20 replicate Skeeter Buster simulations. This statistic is calculated (1) for the number of pupae within a house, noted *L_w_*(*d*), and (2) for the houses themselves, being non-randomly distributed, noted *L*(*d*). Here we plot the difference between the increment in *L_w_*(*d*) and the increment in *L*(*d*) – that is, (*L_w_*(*d*) *- L_w_*(*d-1*)) *–* (*L*(*d*) *- L*(*d-1*)) for all values of *d*. The distance *d* between two houses in this model is defined as the number of steps (horizontal or vertical only) separating these two locations in the grid. The existence of a significant cluster of size *d* is marked by a positive value of this difference, while a value of 0 is expected under random distribution.

We compute the values of the spatial statistics used in the previously mentioned study [20] to characterize the potential clusters in the simulated population. First we calculate *L_w_*(*d*) to provide a global measurement of the level of clustering in the number of pupae produced per house in our simulated population. We show that no significant clustering is observed, even at the smaller scales (Figure 5). This corresponds to the results in the empirical analysis [14].

**Figure 6:**
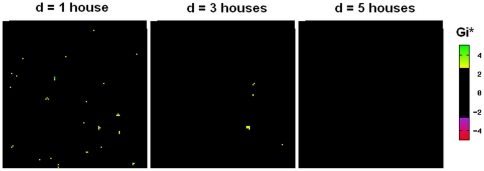
Identification of individual houses in the simulated population as members or non-members of pupal clusters. Getis' *G_i_* values are calculated for each house at distances 1, 3 or 5 houses. Significant positive values of *G_i_* (yellow to green) indicate members of a positive clustering of pupae (grouping of high numbers). Significant negative values of *G_i_* (purple to red) indicating members of a negative cluster (grouping of low numbers) are not observed in this setup. Houses that are not identified as members of either type of cluster are shown in black. Calculations with *d* >5 houses reveal no clusters, positive or negative (not shown).

Finally, we calculate local statistics *G_i_*(*d*) [20], [39] to identify each individual house in our simulated area as being a member or non-member of clusters of size *d* for the number of pupae per house. We show that clusters of small sizes can be found in our simulated population (Figure 6), consistent with the notion that houses vary in their productivity in terms of numbers of pupae. However, these clusters are no larger than 4 houses wide (Figure 6), again consistent with the observed absence of clustering at scales much larger than a household in Iquitos [20]. The absence of these larger clusters suggests that there is no spatial correlation in the productivity of individual households; additionally, it reveals that highly producing individual households are not sufficient to constitute a cluster larger than 4-house wide, consistent with the notion that dispersal of *Ae. aegypti*, at least in Iquitos, is limited to small distances [20].

### Application to Buenos Aires

For the Mataderos neighborhood of Buenos Aires, Argentina, Skeeter Buster was customized using only local weather data [23]. The distribution of containers per house was taken from the Iquitos data. The overall food input in those containers is adjusted to match the observed fraction of positive ovitraps in the study area. This is done by adjusting the coefficient *F*_0_ to 1.5x its value in Iquitos. Since this upward adjustment results in higher amounts of food available for larval cohorts, higher midsummer densities of adults are predicted in the Buenos Aires simulations than in Iquitos. Direct data on adult mosquito abundance would be required to confirm this prediction.

**Figure 7:**
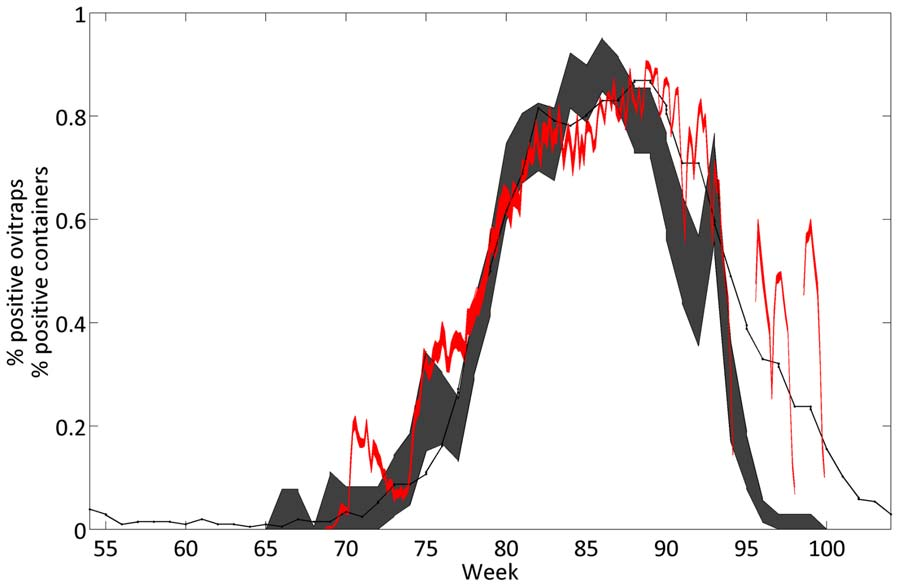
Application of Skeeter Buster to the Mataderos neighborhood in Buenos Aires, Argentina. Time series presented here are from the beginning of July 2001 (week 52) to the end of June 2002 (week 104). Shaded dark gray area: observed fraction (95% CI) of positive ovitraps in a weekly field monitoring [40], [41]. Black line: outcome of Otero *et al.* [12] stochastic spatial model. Shaded red area: Skeeter Buster simulation results (95% CI of 20 replicated simulations) using container data obtained from Iquitos (see text). Discontinuities in the red area correspond to weeks during which no positive container was observed in the model.

We present the outcome of simulations from Skeeter Buster compared to field data as well as to the outcome of another stochastic spatial model by Otero *et al.* [12] (Figure 7). The time series of ovipositions into containers in Skeeter Buster is in good accordance with the observed data from the field, although some discrepancies appear. The most notable difference occurs at the end of the summer (weeks 93–100) when Skeeter Buster predicts significant oviposition events that are not observed in the field. Interestingly, Otero *et al.* [12] observed a similar discrepancy between predicted and observed dynamics using their model of *Ae. aegypti* populations.

## Discussion

In this study, we detail the process through which the Skeeter Buster model can be customized to simulate a population of *Ae. aegypti* in a given location and environmental setting. The level of spatial and environmental detail incorporated into Skeeter Buster makes it possible to develop this type of location-specific application, an important requisite for the ability to simulate the outcome of vector control programs in a given area.

In the Iquitos case, in which the model is set up with detailed ecological information collected from empirical studies in the city, we show that the results of the model are in good accordance with the observed data from the natural population. The remarkable amount of data that were available from this location was helpful not only for testing our model predictions (particularly for spatial analyses), but also for the process of customizing the model to this particular setting, that ensures the ability to faithfully simulate this mosquito population. In particular, as discussed in the Methods, an important part of the customization process is the calibration *a posteriori* of the population levels predicted by the model, based on the pupal counts observed in the field. This is forced by the explicit simulation of the dynamics of within-container nutritional resources in Skeeter Buster, a quantity for which no direct field quantification is available. Although we based this adjustment on a calibration set distinct from the simulated area, this represents nonetheless a less than ideal way to test model predictions. Stage specific numbers for other mosquito life stages can be examined, but collection of accurate data for these life stages (adults, larvae, eggs) is significantly more challenging than for pupae.

The ability to apply a complex model like Skeeter Buster to multiple geographic locations with different environmental and ecological conditions is obviously desirable. We focused our first application study to the Iquitos case that arguably offers the most detailed house-by-house longitudinal entomological data available for *Ae. aegypti*. To test the model's versatility, we applied Skeeter Buster to the Mataderos district of Buenos Aires, a city with a temperate climate. In this case the location-specific customization process was limited to two adjustments: (i) using weather data from Buenos Aires, and (ii) adjusting the overall population levels (measured in the field by monitoring deployed ovitraps) by increasing the daily input of food in containers of all types. Because of the lack of data regarding breeding site distribution and productivity, we carried over the distribution and customization that was established from Iquitos data. This application to Buenos Aires should therefore not be regarded as an effort to provide accurate predictions regarding all aspects of population dynamics in this particular location, but rather as a test of the model's reliance on specific input data.

This exercise shows that Skeeter buster, even with this limited calibration, can capture the temporal dynamics of population expansion and decline across one year under a temperate climate like that of Buenos Aires. Specific discrepancies between observed and simulated time series show, however, that the predictive ability of the model is limited for this location when population levels begin to decline. This could be evidence of an inappropriate parameterization of the model. In our default settings, for example, the minimal water temperature for egg hatch is set to 22°C. Although this value may be appropriate for an equatorial location, it is likely that hatching can occur in colder water at more temperate latitudes (H. Solari, pers. comm.) More generally, it should be noted that the ability to replicate one type of time series at the population scale does not demonstrate the ability of Skeeter Buster to capture other details of the population structure, like actual stage-specific numbers or spatial distribution.

By contrasting the Iquitos and Buenos Aires simulations presented in this study, we illustrate the relationship between Skeeter Buster's ability to simulate specific aspects of *Ae. aegypti* population dynamics and the requirements for specific input data to parameterize and calibrate this complex model. If the model is to be used to simulate and/or predict the temporal profile of average population levels in a given location, then our application to Buenos Aires demonstrate that the data requirements to obtain satisfactory predictions are relatively inexpensive: location-specific climatic data are sufficient. In that case, many aspects of Skeeter Buster's complexity appear superfluous, particularly the detailed spatial distribution of houses and breeding sites. This is consistent with the idea that spatial heterogeneity does not affect average mosquito numbers in Skeeter Buster as shown in a previous study [13]. This is also illustrated here by the match observed in Buenos Aires between field data and simulations run with container data obtained in Iquitos (whereas it is likely that the actual distribution, types and productivity of breeding sites in Buenos Aires differ greatly from that of Iquitos). In other words, the simulations presented in this study demonstrate that detailed container information such as that collected in Iquitos is not necessary to simulate a temporal profile of *Ae. aegypti* numbers, and an ecologically unrealistic container distribution is sufficient in this case. In fact, a simpler distribution can be used with the same results, and we investigate this question further in a separate study by taking a model comparison approach between Skeeter Buster and a model that does not include such detail at the container level [12].

In many instance, knowledge of the temporal profile of average numbers of mosquitoes would, however, be insufficient. For example, heterogeneity in mosquito numbers among houses is an important factor in many aspects; most notably, it impacts the efficiency of control strategies as well as the dynamics of disease transmission by adult vectors [20], [35]. Similarly, at the container level, differential productivity of various container types is crucial information to design effective control programs. The ability of a population dynamics model to capture these details is therefore necessary if this model is to provide guidance for control programs in a given location. In that regard, the Iquitos case study presented here demonstrates Skeeter Buster's ability to operate on this level of detail, thanks to specific aspects of this model's complexity that, in this case, are an integral part of this particular ability. Importantly, this is also contingent on the availability of detailed data on container ability and distribution among houses.

This comparison illustrates the advantages and limitations of using a complex model like Skeeter Buster to simulate specific *Ae. aegypti* populations. The ability to incorporate the environmental and ecological specificities of the location of interest make Skeeter Buster a very adaptable model, a trait that is particularly important in order to guide the development of control strategies that are optimized for a specific location. Yet this level of specificity can only be achieved if the model can be properly customized and its predictions evaluated, which requires the availability of detailed data on the field population. We suggest that the steps presented here could be replicated to apply the model to other locations, insofar as the essential data is available, and keeping in mind that the data requirements are themselves contingent on the level of detail required in the simulation results. Information on the weather-related variables (temperature, precipitation, humidity) are easily available for most locations worldwide from the source used here [23], and provide a first level of location-specificity for Skeeter Buster (as illustrated by our Buenos Aires simulations). If simulating finer details of the temporal and spatial dynamics of a particular mosquito population (as done here for Iquitos) is of interest, extensive field surveys, like pupal/demographic surveys [43], [47]–[49], are needed, particularly collecting data on the local distribution of water-filled containers and their relative contribution to the population of adult mosquitoes.

Overall, the process by which a complex mechanistic model like Skeeter Buster can be evaluated and used with confidence is a conceptually and practically complex task [50]. This process is typically referred to as “model validation”, an essential but controversial part of the development of useful modeling tools [51]. Whether or not a model can ever be fully and definitively validated is a debate beyond the scope of this study. In the case of predictive ecological models, validation is generally recognized as the ability to give reliable and robust predictions regarding a given set of biological questions [52]. Specific approaches have been identified to achieve this objective [53] and our approach for the evaluation of Skeeter buster was designed accordingly. The type of study presented here, a retrospective analysis based on existing data, is naturally not sufficient to establish the validity of this model in any specific location. However, it provides a valuable test with regards to model evaluation, examining how model predictions can withstand falsification efforts. The concordance between simulated and real population dynamics observed in the two case studies presented here constitutes a necessary first step in evaluating whether Skeeter Buster can provide such predictions.

To further establish Skeeter Buster's validity and robustness with confidence, additional studies of this model's predictions will be required. In particular, it is important to recognize that this study was limited to prediction of dynamics of an unperturbed mosquito population. If the model is to be used to predict the outcome of high-intensity vector control strategies, the ability of Skeeter Buster to predict the dynamics of a perturbed population, notably in response to control measures, must be further demonstrated. In that case, prospective studies, based on controlled field experiments monitoring population dynamics after a given type of intervention, will be especially informative.

## Supporting Information

Figure S1 Selection procedure for extended simulation set. + markers represent individual properties in Iquitos. Green: 153 houses constituting the original simulation set (see shaded area in Fig. 2). Red circles: additional houses that, together with the original 153 houses, constitute the extended simulation set.
(TIF)

Figure S2 Distribution of the number of visits per house in the original selected set of 153 houses (gray) and in the extended set of 612 houses (red).
(TIF)

Figure S3 Upper panel: time series comparison with various compositions of the simulated area. **A** (**black**): 153-house simulation set replicated 4 times (setup used in the main text). **B** (**blue**): same 153-house set simulated once, i.e. not replicated. **C** (**red**): 612-house extended simulation set (see Fig. S1) simulated once. Note that the time series for treatments A and B (black and blue lines) match very closely and are therefore hard to distinguish. Lower panel: average and standard deviation of the total number of pupae in the simulated area across 2 years of simulation (years 2 and 3, after 1 year burn-in).
(TIF)

Text S1 Selection of simulation set and effects of set replication.
(DOC)
