# Supplementary figures and images for: Evaluation of Location-Specific Predictions by a Detailed Simulation Model of Aedes aegypti Populations
Source: PLoS One. 2011 Jul 25;6(7):e22701. doi: 10.1371/journal.pone.0022701 (PMC3143176; doi:10.1371/journal.pone.0022701)

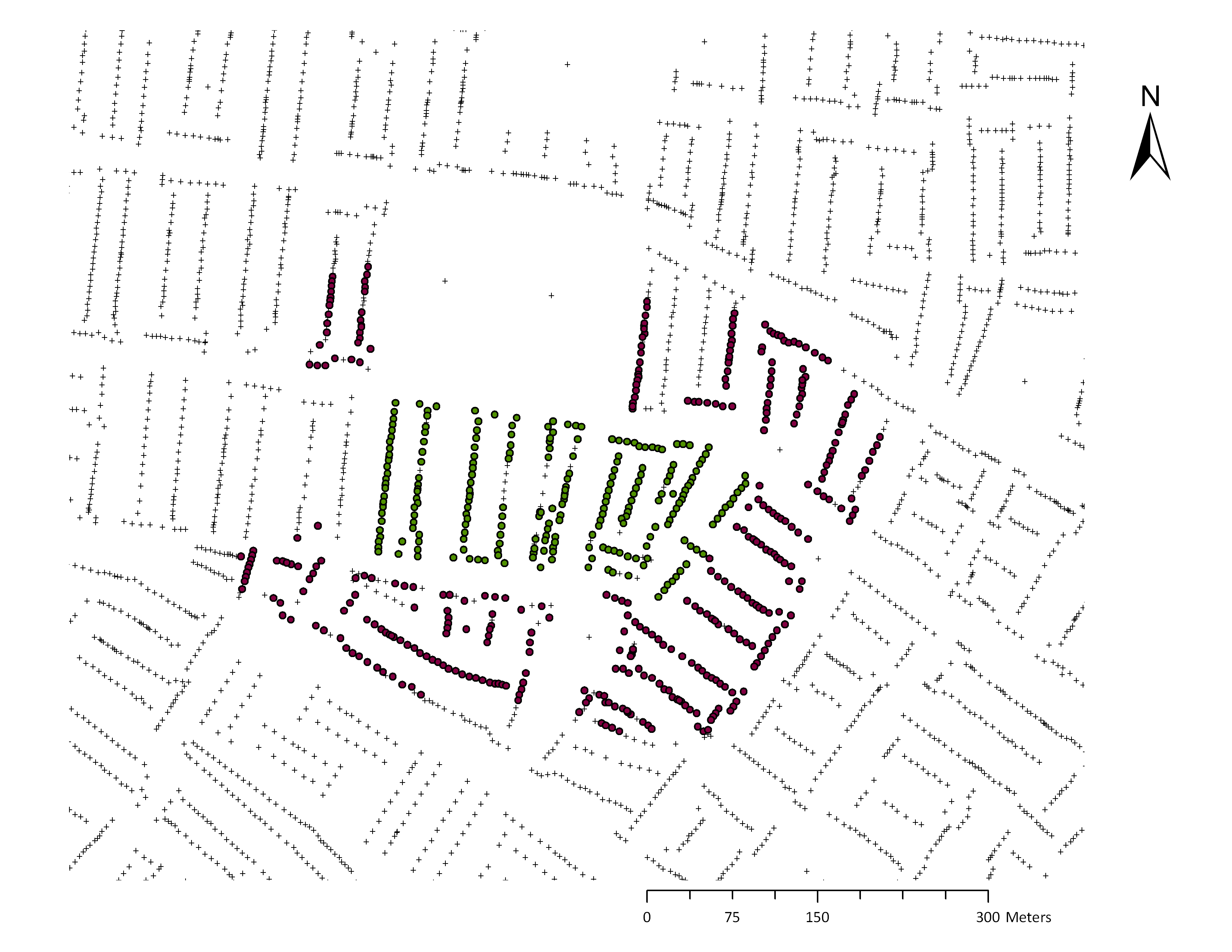

Supplement: Figure S1 — Selection procedure for extended simulation set. + markers represent individual properties in Iquitos. Green: 153 houses constituting the original simulation set (see shaded area in Fig. 2). Red circles: additional houses that, together with the original 153 houses, constitute the extended simulation set. (TIF) [file pone.0022701.s001.tif]

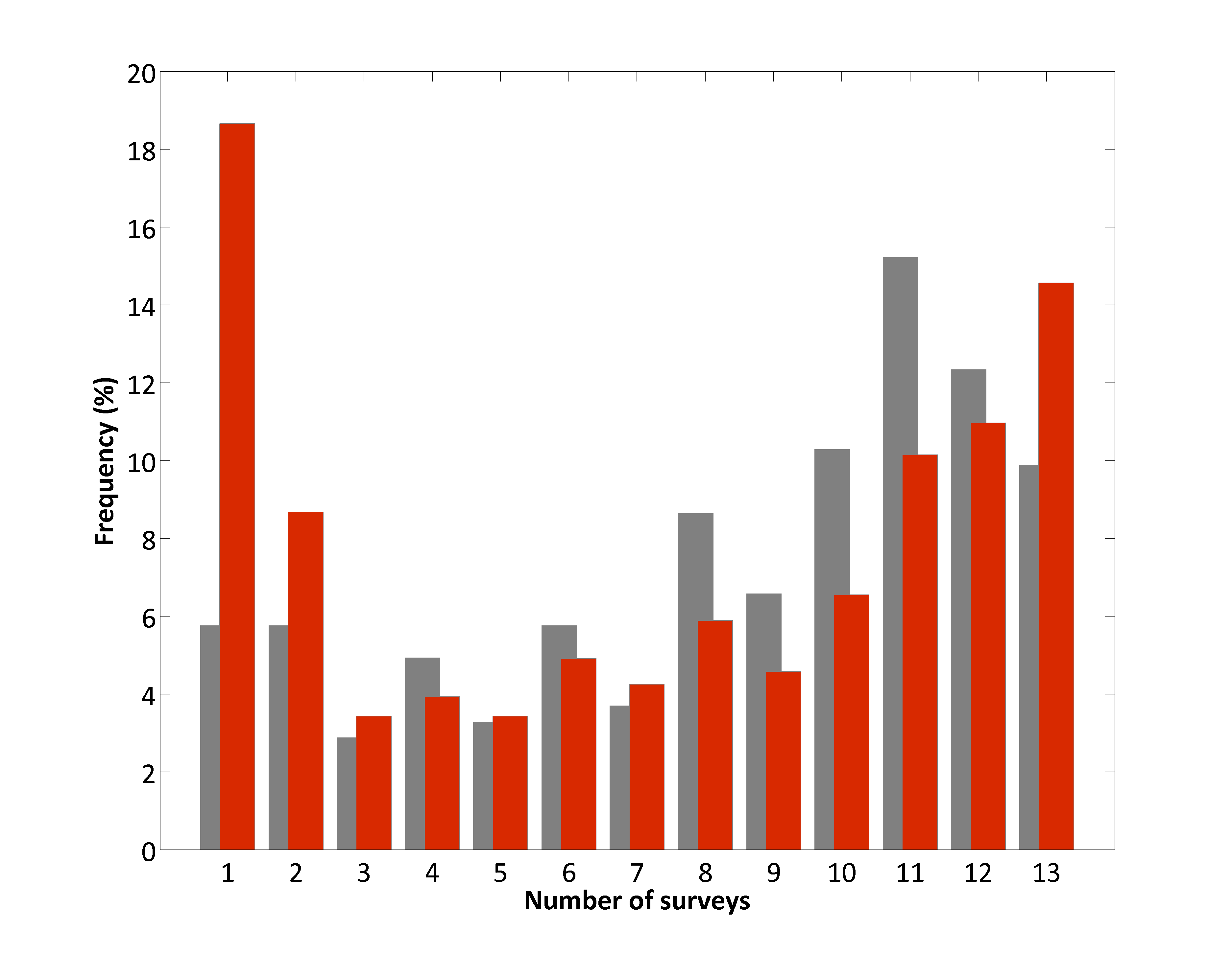

Supplement: Figure S2 — Distribution of the number of visits per house in the original selected set of 153 houses (gray) and in the extended set of 612 houses (red). (TIF) [file pone.0022701.s002.tif]

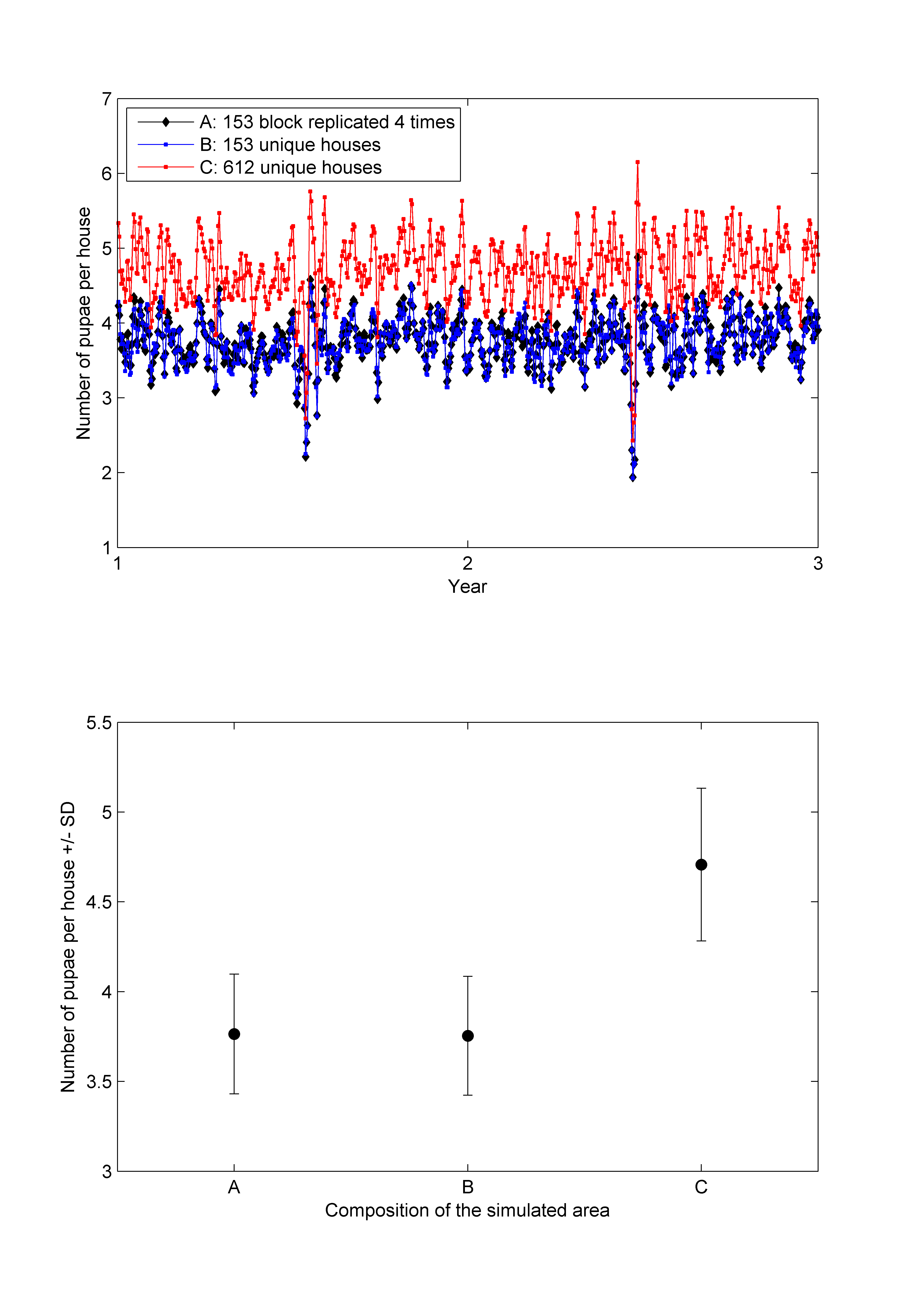

Supplement: Figure S3 — Upper panel: time series comparison with various compositions of the simulated area. A (black): 153-house simulation set replicated 4 times (setup used in the main text). B (blue): same 153-house set simulated once, i.e. not replicated. C (red): 612-house extended simulation set (see Fig. S1) simulated once. Note that the time series for treatments A and B (black and blue lines) match very closely and are therefore hard to distinguish. Lower panel: average and standard deviation of the total number of pupae in the simulated area across 2 years of simulation (years 2 and 3, after 1 year burn-in). (TIF) [file pone.0022701.s003.tif]
