# Supplementary material for: Evaluation of Location-Specific Predictions by a Detailed Simulation Model of Aedes aegypti Populations
Source: PLoS One. 2011 Jul 25;6(7):e22701. doi: 10.1371/journal.pone.0022701 (PMC3143176; doi:10.1371/journal.pone.0022701)
Supplement: Text S1 — Selection of simulation set and effects of set replication. (DOC) [file pone.0022701.s004.doc]

**Supporting Text S1:**

**Selection of simulation set and effects of set replication**

In the main text we describe the selection of 153 houses as the basis for our simulation set. For the analyses carried out in this study as for many other situations, it can be desirable to extend the simulated area beyond 153 houses.

In this study (main text) we chose to do so by replicating our basic subset of 153 houses to construct a larger simulated area. This process was motivated by the purpose of confronting simulation results to observed data, and therefore by the need to focus our study on a heavily surveyed region of Iquitos to maximize the reliability of the collected field data.

Here we investigate the consequences of this replication by examining alternative setups for our simulation area.

**Construction of alternative selection sets**

In this section we consider three possible constructions of our simulated area by selecting different houses from the set of visited households in Iquitos.

Set A (612 houses): the set used in the main text of this study: our basic 153-house subset replicated four times;

Set B (153 houses): the basic 153-house subset, not replicated;

Set C (612 houses): a selection of 612 unique houses from the set of visited households in Iquitos.

To establish simulation set C, we selected 612 houses from the ensemble of visited houses in Iquitos, by choosing those visited houses that are located closest to our original set of 153 houses (Figure S1). Note therefore that set C includes these 153 houses, as well as 459 other houses not included in the original simulation set.

**Survey frequency distribution of the three simulation sets**

The 153-house set used in the main text, and the basis of sets A and B here, was selected for its relatively high survey frequency (see Fig. 1 for comparison with the entire Iquitos area). Accordingly, by extending our selection to 612 houses around this set, we constitute a new set (set C) whose survey frequency distribution is shifted towards a lower number of surveys (Fig. S2). Note in particular the overrepresentation in set C of houses surveyed 3 times or less, and the underrepresentation of houses surveyed 8-12 times. On average, the information collected at the house level in set C is therefore expected to be less reliable than that in sets A and B.

**Comparison of time series based on the three simulation sets**

We run Skeeter Buster with each of the three sets, in a fashion similar to that detailed in the main text (environmental data from Iquitos, 1 year burn-in and the 2 following years analyzed). Results from these simulations confirm that sets A and B give very similar results in terms of number of pupae per house, both for average numbers and temporal variation (Figure S3; temporal coefficient of variation in number of pupae per house among 2x365 simulation days are 8.85% and 8.82% for sets A and B, respectively).

Simulations using set C give a different result for the average number of pupae per house. This is expected, since set C has a different container composition, both in numbers and suitability (i.e. food availability). More precisely, while set A is composed of 3,484 containers, set C is composed of 3,695. The overall suitability of these sets of containers can be estimated by the sum of daily food gain values *F* (see eqn. 1 in main text) of all containers in the simulated area. This sum is equal to 2628.3 mg (liver powder equivalent) for set A and 3141.7 mg for set C. This higher total availability of breeding sites and nutritional resources is responsible for the higher average number of pupae per house.

Interestingly, however, the temporal variability in the number of pupae per house is not affected by the composition of the simulation set. The temporal coefficient of variation in number of pupae per house is 9.03% for set C, i.e. very similar to what was observed in sets A and B. Additionally it is clear that external factors (e.g. environmental data) play an important role in shaping some of the major troughs in these time series, and affect results from all three sets in a similar fashion.

From a methodological standpoint, the results from a 612-house simulation area appear therefore qualitatively similar, whether the simulation set is composed of 612 unique houses or a 153-house set replicated 4 times. On the other hand, the reliability of data collected from field surveys is negatively affected by extending the house selection outside the most frequently surveyed areas in Iquitos. For the purpose of this study and for comparison of model predictions with data from field surveys, we elected to limit our house selection to a small area, prioritizing the reliability of field collected data provided by repeated surveys.
